# The Education of Dentists

**Published:** 1881-05

**Authors:** 


### THE EDUCATION OF DENTISTS.

BY A PHYSICIAN.

WITHIN the memory of many now living, the barbers and blacksmiths in town, and the blacksmiths, local preachers and horse trainers in the country, in common with practitioners of medicine, gave the teeth the surgical attention they received. And as dental surgeons, these various classes held about equal rank in the popular mind, and really differed but little in merit, in their treatment of the teeth, this treatment consisting of twisting them out with the barbarous turn-key, called "pullikins," or breaking or filing off sharp projections, so that they would not wound the tongue or lips. Often has the writer heard warm discussions as to the superiority of the leading physician of his native town and a blacksmith of the same place; and the champions of the blacksmith claimed the victory, inasmuch as the physician patronized him and his turn-key, while the smith either extracted his own teeth or sought the torturing touch of a fellow blacksmith.

A number of years ago, Dr. Watt deposited in the museum of the Ohio Dental College a forceps made by the father of a noted antiquarian, and used, more or less, in extracting teeth for more than half a century—used the last time in removing the last tooth of the maker when he was nearly a century old.

Dental surgery is not such now. Why was it such then? More acute pain than toothache is scarcely known to the race. Decay of the dental organs is more common than any other disease. The extraction of a tooth is one of the most painful operations known to surgery. Decay and toothache are not of recent origin. For a long time they have been as common as flies in summer. Unweaned babies cried with the tortures of toothache, or died in convulsions or coma, from the reflex irritation of dentition. Prospective mothers agonized in the throes of odontalgia, whether there teeth were sound or unsound. Dyspepsia griped and starved its staring victims, because their stomachs could not digest the unmasticated food, mangled, but not chewed, by the rotten, filthy, fetid teeth, which, unable to perform their functions, only defiled and poisoned what they could not prepare for nutrition. This distressed and depressed condition was handed down from sire to son, was visited on even the third or fourth generation, because the stream cannot rise higher than the fountain, and because none can bring a clean thing out of an unclean.

And were all these things known to physicians and surgeons? Were physicians educated then? Had they been educated preceding the induction of this state of affairs? Were there medical schools? And, if so, did they teach the anatomy, physiology, and pathology of the teeth? Did they teach dental surgery? Yea, verily, did they, for the writer heard them. In one of them, in a course of four months, the professor of anatomy and physiology devoted a half hour to the consideration of the teeth, and the professor of surgery gave them still more attention, spending an entire lecture in talking about them and the instruments used in their extraction.

The writer of this is a physician, and from observation, and judging by the dental attainments of his fellow practitioners of medicine, he is led to believe that other medical schools gave the teeth but little, if any, more attention than was given by his *alma mater*. But, in view of the fact that the physicians of that day, and especially the college professors, were well read and wide-awake on many, if not most of the ills of human life, how could there be such neglect of the dental organs? The answer is easy.

Notwithstanding the intense agony, toothache is not apt to kill. Defective teeth do much to shorten life, and Sir Thomas Watson offers as a prominent item in the causes of increasing longevity, the better condition of the teeth, brought about by the revival and development of dental surgery. Indigestion, and the consequent defective nutrition, shorten life, and the physicians of the period of greatest neglect were well aware of this, but as the results were not so immediate and direct, their attention was taken up by the ills which threatened sudden or early danger. In consequence of this state of feeling, although medical schools were thoroughly organized, and well manned, dental surgery made no progress worthy of the name, and, under similar circumstances, could make none worthy of the name. The ills which flesh inherits are too many, too serious, and too grave in their results, for consideration in a college course, as then laid down by the popular views of medical education, to give time and attention to toothache, and the troubles of dentition, which these call for. But a change was coming.

A few physicians began to give their undivided attention to the mouth and its troubles. These were lonesome and sought company. They persuaded others to follow their example. These led others, and associated efforts resulted. In society, where men compare their mental attainments with those of their comrades, a spirit of emulation arises. They are not content with present acquisitions, and hence seek for more and greater. A necessity for instruction is thus demonstrated. A school becomes necessary and is established. It is devoted to specific instructions, because it originated in specific want. If a large school, or if it includes a wide range of instruction in its curriculum, it may be called, appropriately or otherwise, a college. Some such train of circumstances led a few physicians, step by step, through a long process of development, resulting in the establishment of a dental college.

This was a new thing under the sun, and its career was hopefully, fearfully, jealously and sneeringly watched. It lived and grew, and quiet looks of calm contentment, and smiles of joy and comfort took the places of frowns and looks of despair and woe. In the first ten years of its existence it did more for the progress of dental science than all the medical schools had done from the dawn of civilization. It soon had company. Other dental colleges were organized, and a new feature of professional education was thus fairly inaugurated, and thus came the revival, or rather the creation of dental science referred to by Sir Thomas Watson, as noticed above.

In the formation of dental colleges, physicians took the lead, indeed did all the work. Nor did they forget that they were physicians, but in organizing their special schools, they laid the foundation broad and firm on the basis of medical science. As in medicine, they recognized anatomy as the chief corner stone, and chemistry as the bond uniting, and practically utilizing, all the sciences included in the curriculum of study. And in few medical colleges have better arrangements been made for the study of these than those provided in the leading dental schools. They also made ample provision for the study of pathology and therapeutics, and the attainments of their pupils in these directions compare favorably with those of the students of cotemporary medical colleges. There is but little doubt that the colleges have done more to advance dental science than all other agencies combined, and, in general, their course is still upward as well as onward.

After the great advantage to be derived from dental colleges had been fully recognized, the restlessness incident to the human mind, especially to American mind, began to manifest itself, and to inquire if there might not be devised a more excellent way; and this inquiry was certainly commendable. Some advocated the establishment of a dental chair attached to medical colleges. That is, a professor of dental surgery was to be added to the faculty of each medical college, and this was to elevate dental surgery, as the medical students would thus become dentists. The plan is a good one, not to make dentists, but to give young physicians a better knowledge of the teeth and their relation to morbid states, than they would otherwise get. Physicians are demanded in villages and country places that cannot each support a dentist.

It is well for the physician and his patrons that he can, at least, diagnose dental diseases in such cases. A professor of, or lecturer on, dental surgery in a medical school, can be of very great use to the medical students, and, through them, to the community at large. But for a preparation to practice dentistry the plan is futile. As well might we expect the medical students to become blacksmiths by an occasional lecture on metallurgy, or ministers of the gospel after a few lectures on biblical literature intermingled with their regular course.

It is very desirable that dentists should have a thorough medical education, yet there are certainly difficulties in the co-education of medical and dental students. For instance, it is highly important that both should study carefully the anatomy of the whole human system, yet as time is precious, and human memory unretentive, the medical student should give extra attention to certain parts, such as may be involved in operations for hernia and the like, while dental students should give just as careful attention to certain other parts, as the face. This difficulty lies equally with chemistry, pathology, therapeutics, etc. And herein lies all the objection we see to dental colleges in connection with universities, where the same professor, and mainly in the same lectures, teaches both classes of students.

Universities often have special advantages in the way of museums, cabinets, and chemical and philosophical apparatus, that may more than atone for the want of special directness in their lectures. Of course neither class of students can hear or learn anything that is not valuable, but as their memories cannot retain all that they are taught, and the time alloted in ordinary courses of study is too short to teach minutely all the details of any science recognized as part of the course, the lack of special application in the lectures must be a misfortune. However, this can be overcome mainly by a little additional labor on the part of the teachers, and it is quite propable some such method is practised.

The education of the hands and eyes is a very large and important part of the dental education. The hand of the mechanical genius, and the eyes of the artist, are good factors to start with in making a dentist. A good, strong, well balanced mind is the all important requisite here, as in all other professions, but the other qualifications are very important. In the popular mind they are possibly overestimated, however, as I was told by a dentist, aged and experienced, that when asked to take a private student, the parent's or guardian's recommandation is, invariably, "He's a perfect genius—always tinkering with tools." But, on the other hand, the dentists may estimate them too low, as I think the dentist referred to did, when he told me he could always train the hands without trouble, while the writer has seen hands that could not be trained to handle tools accurately.

It seems at first thought, that it should not be more difficult to prepare to practice dentistry, than to practice medicine. But, if we take the view that dentistry is a specialty of medicine, and place the dentist on the same platform with the oculist, the case is quite different. In that view, the dentist must take a full medical course, and receive the degree of M. D., before he is ready to begin the study of dentistry. In other words, double the time will be required to make a dentist that is necessary to make a physician. It is propable a majority aiming at dentistry will prefer to consider it an independent profession, and seek by a direct road to reach it, rather than take the double track, merely for the sake of medical recognition. It matters but little after all about the standing or recognition, provided the attainments are made.

But there are two sides to the question of recognition. If the dentist must take the general medical course, and get the M. D., before he is recognized as of the fraternity, should not the physician take the special dental course, and get the D. D. S. before he is qualified to extract or otherwise interfere with the teeth? Of his brother physicians, the writer has seen regular graduates extract the first permanent molars (called the six-year molars), in mistake for temporary teeth. Such maiming is simply outrageous, and indicates that its perpetrator needs dental, as much as any dentist needs medical instruction.

But in the way of dental knowledge there is a revival in both professions. In a number of the medical schools, dentists are teaching, and the physicians thus taught will not make serious mistakes, like that referred to above, and the dental colleges, in connection with the universities, and those independent, will stimulate each other, and a generous rivalry cannot be otherwise than promotive of good results.

It is not the aim of the writer to determine which of the present methods of dental education is preferable. Both are good. If by pointing out some of the advanatages and drawbacks of each, the writer can awaken closer attention to the education of both physicians and dentists, he will feel amply compensated for this hastily written article.—*Ohio State Journal of Dental Science.*

